# High precision laboratory dryer for thin layer and bulk drying with adjustable temperature, relative humidity and velocity of the drying air

**DOI:** 10.1016/j.ohx.2020.e00133

**Published:** 2020-08-18

**Authors:** Sebastian Reyer, Sebastian Awiszus, Klaus Meissner, Joachim Müller

**Affiliations:** Institute of Agricultural Engineering, Tropics and Subtropics Group, University of Hohenheim, Garbenstraße 9, 70599 Stuttgart, Germany

**Keywords:** Postharvest technologies, Precision drying, Sorption isotherms, Drying behaviors, Moisture ratio, Drying curve, Thin layer dryer

## Abstract

Convective hot air drying is one of the most common post-harvest processes within the agricultural sector and a standard process in the food industry. The technique is used on fruits and vegetables in order to increase their added value or to extend their shelf life. Drying is highly dependent on the condition of the drying air in terms of temperature, relative humidity and velocity and on the thickness of the product layer. The influence of these drying parameters on the drying behavior and quality is product-specific and has to be investigated in laboratory experiments due to the high costs for full-scale evaluation. For this purpose, a high precision laboratory dryer was developed in order to achieve controlled and stable climate conditions during the drying of light bulk material.

To avoid temperature drift of the load cell during drying, a temperature-controlled sensor housing was applied. To further stabilize the signal, it was corrected with the instantaneous temperature.

The high precision laboratory dryer HPD TF1 could be potentially useful to establish drying curves for defined temperature, relative humidity and velocity of the drying air. Further potential applications are the establishment of sorption isotherms or the determination of diffusion coefficients of various materials.

## Graphical abstract

**Figure:**
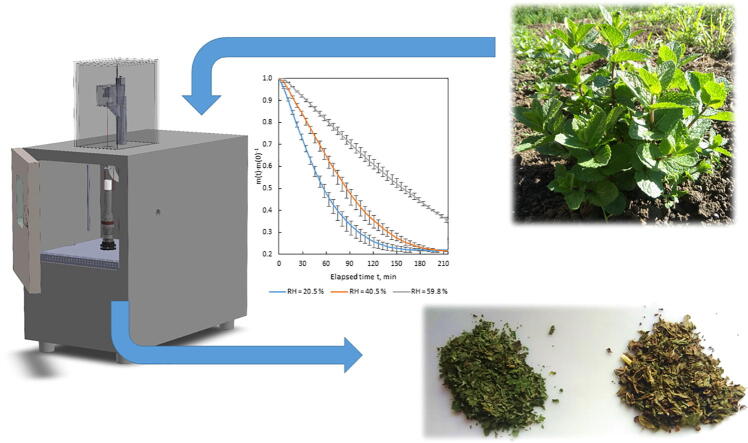

## Nomenclature

**Greek letters**
η: Dynamic viscosity Pa∙s
ϱ: Density $\mathrm{kg}∙m^{-3}$
Δ: Difference of values

**Notations**
f: Frequency %
i: Variable
n: Number of values
m: Mass
MAPE: Mean absolute percentage error %
P: Air pressure kPa
$R^{2}$: Correlation coefficient
Re: Reynolds number
RH: Relative humidity %
T: Temperature *°C*
t: Time s
u: measuring uncertainty
U: Voltage V
v: Velocity $m∙s^{-1}$
X: Water content
Y: Value
Z: *A*ltitude m

**Subscripts**
0: Standard pressure at sea level
average: Average value
cal: Calibration value
calc: Calculated value
corr: Corrected value
dew: Dew point value
meas: Measured value at time point
max: Maximum value
norm: Normalized value
p: Altitude constant
set: Set value
w,sat: Water vapor pressure at saturation

Specifications table

**Table:** 

Hardware name	HPD TF1 – High Precision Dryer Through Flow 1
Subject area	• Engineering and Material Science • Food Science • Pharmaceutical Science • Biological Sciences (e.g. Microbiology and Biochemistry) • Agricultural Science
Hardware type	• Measuring physical properties • Measuring drying behavior
Open Source License	CC BY 4.0
Cost of Hardware	Approximate cost of hardware 40,000 €.
Source File Repository	https://doi.org/10.17632/g9w6ct4cgk.3

### 1 Hardware in context

Convective hot air drying is one of the most common post-harvest processes within the agricultural sector and a standard process in the food industry. It is widely applied on staple crops like cereals and legumes to achieve stable conditions for safe storage. Furthermore, the technique is used on fruits and vegetables in order to increase their added value or to extend their shelf life [1]. Due to the increasing interest in bioenergy, hot air drying is also applied on biomass like wood chips or biogas digestate in order to improve the heating value or the transportation value, respectively [2]. Depending on the type of the drying product and the available energy source, different drying techniques like belt-, drum-, feed-and-turn- or solar dryers are used, where heated air is passed through the drying material [3]. Drying is highly dependent on the condition of the drying air in terms of temperature, relative humidity and velocity and on the thickness of the product layer [4], [5]. The influence of those drying parameters on the drying behavior and quality is product-specific and has to be investigated in laboratory experiments because an evaluation in full-scale dryers is too expensive. For this purpose, various types of laboratory dryers have been developed to simulate the drying conditions in a full-scale dryer as good as possible.

Both drying ovens as well as the climate chambers can be used for laboratory drying experiments. In this case the temperature is adjustable [6], [7] and - in the case of climate chambers - also the relative humidity of the drying air. The drying process is based either on natural convection or on forced ventilation of the entire chamber, but the air is not systematically forced through the drying product layer [8].

Overflow dryers show a good performance if used for single layer drying as for fruit or vegetable slices [9]. In contrast, overflow dryers are less suitable for drying bulky materials like dewatered sewage sludge or digestate. These products tend to build up crusts during drying, which reduce the drying rate and lead to uneven drying. In overflow dryers, the drying air is overflowing the product layer above and/or below, while in through-flow dryers the drying air is passing through the bulk or product.

The mass loss during drying can be determined by weighing the product either manually [10] or automatically [11]. Independent from the overflow- or through-flow, air circulation in the dryer has to be interrupted during weighing in order to avoid uplift effects and measurement errors.

Flow resistance is influenced by the shrinking of bulk materials during the drying process. If the ventilation fan is set to a fixed speed, the through-flow velocity will change in accordance to the flow resistance. In most cases, air velocity is set at the beginning of the experiment by choosing the rotational speed of the fan or by a valve, controlled by a heat wire anemometer [12], [13]. In order to achieve a stable airflow velocity through the product, the air velocity has to be measured continuously during the drying process and the speed of the fan has to be controlled, respectively.

The high precision laboratory dryer, presented in this study, was developed in order to achieve controlled and stable climate conditions during the drying of thin layers or shallow bulks of material.

### 2 Hardware description

A high precision laboratory dryer to simulate convective hot air drying in through-flow mode, named HPD TF1, was build.

The high precision laboratory dryer HPD TF1 is potentially useful for determination of:

• drying curves for defined temperatures, relative humidity and velocity of the drying air of:
  o of thin layers like one layer of fruit slices
  o of shallow bulks like 1 cm of grain or corn adsorption and desorption isotherms
• adsorption and desorption isotherms
• diffusion coefficients of materials
• isosteric heat of materials

**Fig. 1:**
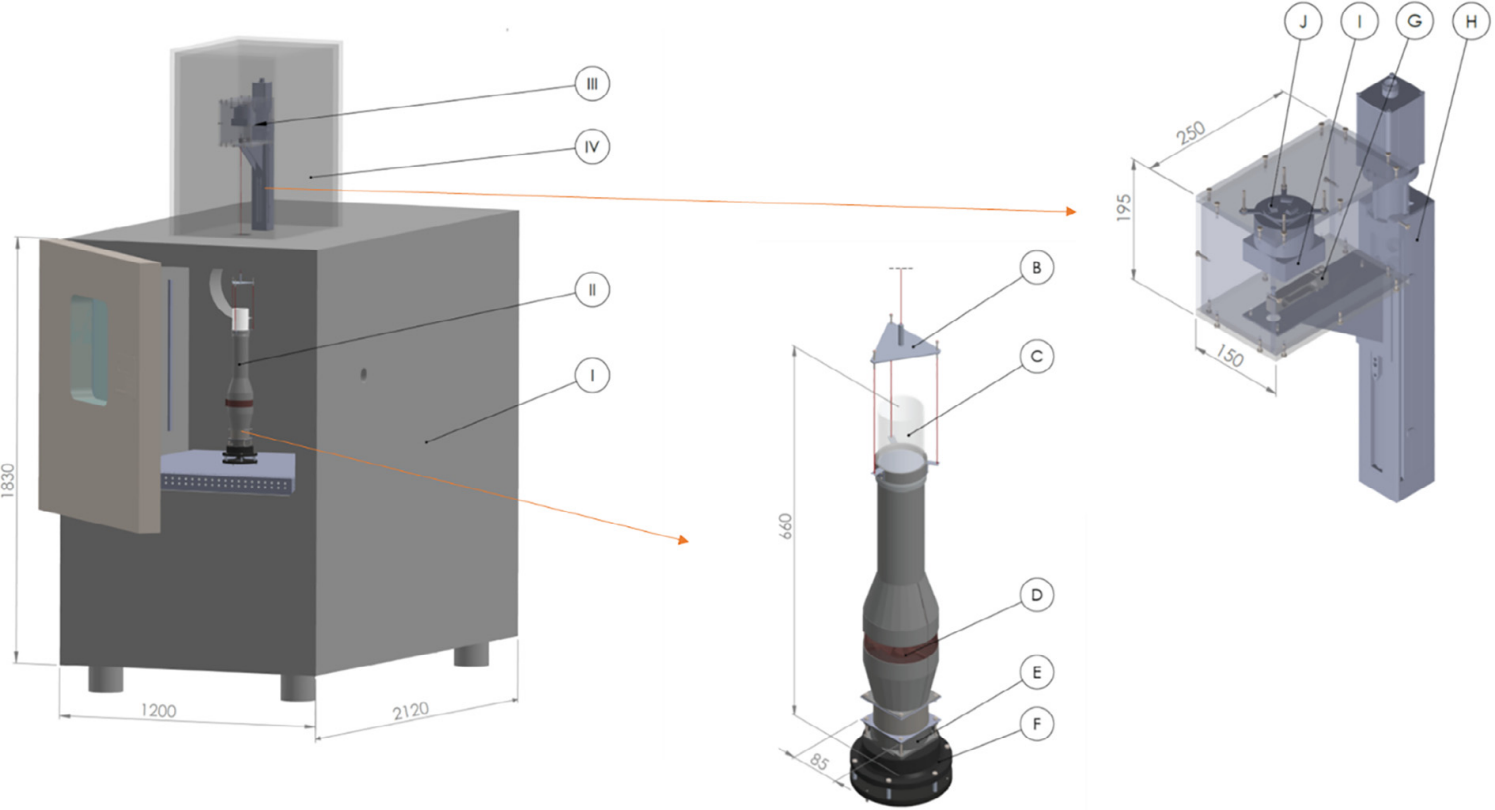
3D-model of the high precision laboratory dryer HPD TF1; (I) air conditioning unit, (II) drying unit, (III) balance unit, (IV) thermal insulation chamber, B Traverse, C Sample tray, D Vane anemometer, E Axial fan, F Radial air outlet, G Balance, H Linear lifting unit, I Heating element, J Axial fan. The views are not on the same scale.

**Fig. 2:**
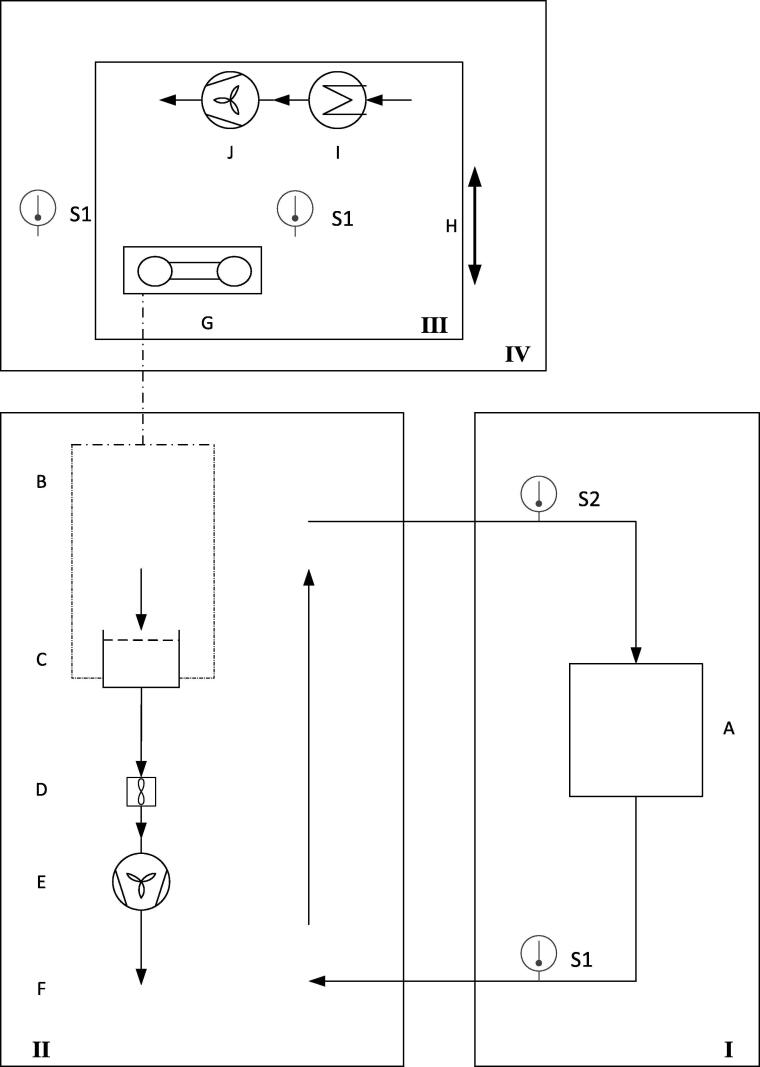
Functional units of the high precision laboratory dryer HPD TF1: (I) air conditioning unit, (II) drying unit, (III) balance unit, (IV) thermal insulation chamber; A Air conditioning, B Traverse, C Sample tray, D Vane anemometer, E Axial fan, F Radial air outlet, G Balance, H Linear lifting unit, I Heating element, J Axial fan, S1 Temperature sensor, S2 Relative humidity sensor.

The HPD TF1 consists of four functional units, which are shown as a 3D-model in Fig. 1 and as a scheme in Fig. 2: (I) an air conditioning unit is based on a commercial climate chamber and provides a constant temperature and relative humidity, (II) a drying unit provides a constant airflow through the drying material, (III) a balance unit performs continuous weight measurements of the samples and (IV) a thermal insulation chamber to protect (III) from rapid temperature fluctuations.

#### 2.1 Air conditioning unit (I)

To provide a constant temperature (T) and relative humidity (RH) of the drying air, a commercially available climate chamber (Type °C −20/1000, CTS GmbH, Hechingen, Germany) was used as basic device for the HPD TF1. The CTS-climate chamber consists of an air conditioning unit (I), which is placed in the back of the actual climate chamber with a usable volume of 1 m^3^ in the front. The air is circulated from the climate chamber through the air conditioning unit by two steel blade ventilators. The fans can only provide a fixed airflow based on their standard rotational speed. In the air conditioning unit, T and RH of the air are adjusted. T can be set between −20 °C and 180 °C and RH between 5 % and 95 %. To realize low RH levels, the dew point temperature (T_dew_) can be lowered by a compressed air dryer to a minimum of −7 °C. Two PT-100T sensors are used in combination with a capacitive RH sensor to control the climate in the chamber. The capacitive RH sensor is part of the climate chamber which is a purchased part. The sensor could be improved by a dew-point RH sensor for a better long term stability.

#### 2.2 Drying unit (II)

**Fig. 3:**
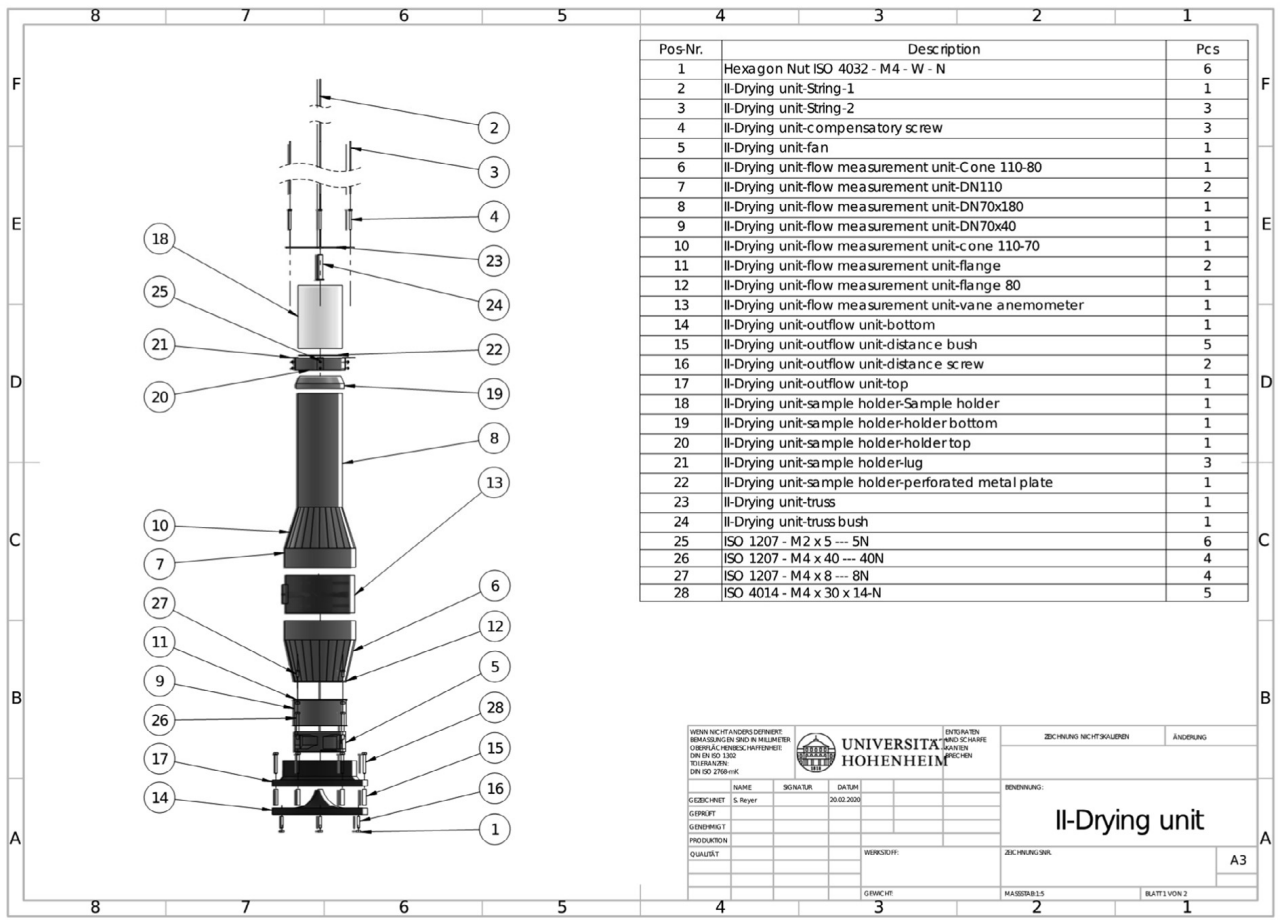
Build introduction for drying unit (II).

A specially developed drying column (II) (Figure 3) is placed in the climate chamber. The drying column consists of a cylindrical air duct with an integrated fan (E), a vane anemometer (D) and a radial air outlet (F). A cylindrical acrylic glass sample holder with an outer diameter of 70 mm and a wall thickness of 3 mm (C) is placed on top of the dryer column. A maximal bulk height of 80 mm represents a sample volume of 257 cm^3^. A steel mesh on the bottom of the sample holder ensures a uniform through-flow of the drying air. The sample holder is suspended from the balance unit (III) via a traverse (B) with a nylon string. The fan (E) (Type 8212J/2H4P, ebm-papst Mulfingen GmbH & Co. KG, Mulfingen, Germany) at the bottom of the drying column sucks the air from top to bottom through the sample holder and the vane anemometer (D) (Type 1468, Lambrecht meteo GmbH, Göttingen, Germany). The vane anemometer is used as a sensor in a control loop to adjust the air speed in the drying column by controlling the speed of the fan. To obtain uniform flow conditions, a honeycomb structure is placed directly above and below the anemometer. With this arrangement, the entire airflow is forced through the measurement device and, therefore, allows recording of the total flow. The radial air outlet additionally contributes to uniform flow conditions in the drying column. A from top to bottom airflow was chosen to prevent floating of the sample in the sample holder at higher air velocities.

#### 2.3 Balance unit (III)

**Fig. 4:**
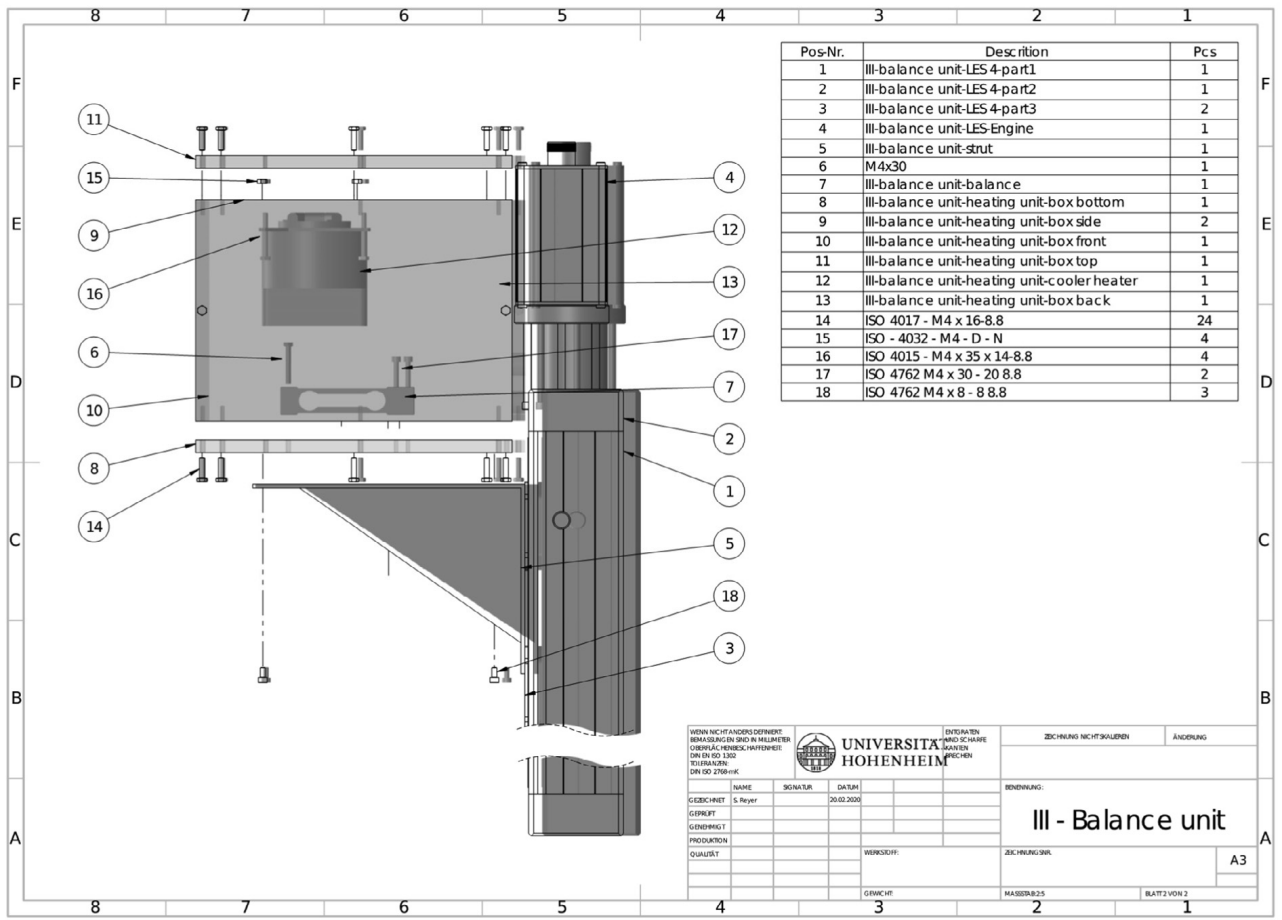
Build introduction for balance unit (III).

A balance unit (III) (Figure 4) is mounted on top of the climate chamber, containing an electronic balance (G) and a heating system (J) to provide stable temperature conditions inside the housing. The balance unit is mounted on an electric spindle drive (H), with which it can be lifted smoothly. The sample holder of the drying column is connected to the balance by a nylon string, which is led through a bore in the ceiling of the climate chamber. Unlike steel, the nylon string has a low thermal conductivity preventing an eventual deposit of water through condensation during the experiments with high air humidity. To weigh the sample within a certain time interval, the sample holder is lifted from the dryer column by raising the balance unit until it hangs freely. Matching cones on the bottom of the sample holder and the top of the drying column ensure an airtight upright position when the sample holder is set back after weighing. During the mass determination process, all fans in the Air conditioning unit (I) and Drying unit (II) are stopped.

The balance (Type AR 0.6 kg, Lorenz Messtechnik GmbH, Alfdorf, Germany) has a measuring range of 600 g ± 0.02 %. It is equipped with a measuring amplifier (Type LCV-U5, Lorenz Messtechnik GmbH, Alfdorf, Germany) with u of ±0.02 %. The spindle drive (Type LES4, isel Germany AG, Eichenzell, Germany) is controlled by a step-controller (Type IT116, isel Germany AG, Eichenzell, Germany). The housing of the balance unit protects the balance from dust and temperature fluctuations. To control the temperature inside the housing, three load resistances (Type RH-5-1%-15R, Vishay Electronic GmbH, Selb, Germany) (J) are connected in parallel to a 12 V source. The electrical circuit is controlled by a metal–oxide–semiconductor field-effect transistor (MOSFET) (Type IRLZ24NPBF, Infineon Technologies, Neubiberg, Deutschland) regulated by a T sensor (Type TS-NTC-103, B + B Thermo-Technik GmbH, Donaueschingen, Germany). A thermal switch is connected in series to prevent the resistors from overheating. The thermal switch disconnects at 75 °C ± 5 °C and reconnects at 65 °C ± 5 °C (Type TK24-T01-MG01-Ö75-S65, Thermorex, Paris, France). The load resistances are mounted on an aluminum plate, which is fixed on a CPU-cooler. In addition, a fan (I) is attached to the cooler, to ensure a fast heat exchange and to circulate the air inside the balance unit. The fan is operated at 5 V to obtain an uniform and smooth air-flow without affecting the measurements of the balance. For temperature measurements, four negative-temperature-coefficient-thermistors (NTC) (TS-NTC-103, B + B Thermo-Technik, Donaueschingen, Germany) are used. Two T sensors measure the temperature within the balance-chamber at different positions; the mean value is used for controlling. In addition, a T sensor is placed on the load resistances to interrupt the heating process at the maximum temperature as set via the software. A further T sensor is used to measure the ambient temperature at the research site.

To measure the voltage of the amplified balance signal as well as the output voltage of the vane anemometer, an USB-6002 I/O device with a resolution of 16-bit and a sampling frequency of 50 kSamples·s^−1^ (Type USB-6002, National Instruments Germany GmbH, München, Germany) was used. For the USB-6002 I/O device u is given with 0.06 % in stable climate condition. Overall u is given with 0.12 % which is used for the calculation. The current signal of the vane anemometer is converted at the 105 Ω shunt resistor to a voltage signal. Furthermore, a voltage output was used to regulate the Pulse-width modulation integrated circuit (PWM-IC) (TL494CN, Texas Instruments Incorporated, Dallas, USA) to control the required fan-speed. The duty cycle is dependent on a reversed DC input signal, which is generated by the I/O device.

#### 2.4 Thermal insulation chamber (IV)

The thermal insulation chamber is made of wood. The plate thickness is 15 mm. A thermal insulation is mounted to the inner walls of the chamber (Armaflex AF Platte 19 mm, Armacell GmbH, Münster, Germany). Furthermore, ventilation openings are drilled into the upper part of the side panels. The natural convection is necessary to remove moisture from the box. The passage of the nylon string from the climate chamber to the thermal insulation chamber (IV) represents a leakage. Due to that leakage the water content in the thermal insulation chamber increases by time. To reduce the water content, this chamber is cooled down and refreshes itself through natural convection.

As communication interface with the climate chamber control system and the spindle drive, a standard RS232 adapter (USB RS232 Interface Cable 2, Wiesemann & Theis GmbH, Wuppertal, Germany) was used. The dryer operation software was programmed using LabView-Software.

### 3 Design files

#### 3.1 Design files summary

**Table:** 

Design file name	File type	Open source license	Location of the file
HPD TF1 2019.EASM	easm	CC BY 4.0	Source File Repository
HPD TF1 2019-SWfiles.zip	zip // Solidworks parts and assembleyfiles	CC BY 4.0	Source File Repository
HPD TF1 2019.STEP	step	CC BY 4.0	Source File Repository
2019-HPD TF1-Installer.zip	Zip	CC BY 4.0	Source File Repository
2020_HPD TF1-electrical circut.pdf	pdf	CC BY 4.0	Source File Repository

HPD TF1 2019.EASM

Assembly file for getting an overview about the construction and operation. eDrawings is a Free 3D viewer of 3DS.

https://www.edrawingsviewer.com/download-edrawings

HPD TF1 2019.zip

SolidWorks-Files with all components for reproduction or improving

HPD TF1 2019.IGS

IGS-Files with all components for reproduction or improving

2020_HPD TF1-electrical circut.pdf

Electrical circuits for the Balance-Heating-Unit and the PWM Control

2019-HPD TF1-Installer.zip

Contains the installer for the Control-Program and also the LabView Runtime-Engine for Microsoft Windows.

### 4 Bill of materials

**Table:** 

Designator	Component	Number	Cost per unit –currency	Total cost -Currency	Source of materials
Climate Chamber	C −20/1000 (CTS GmbH, Hechingen, Germany)	1	€ ~35,000	€ ~35,000	https://www.cts-umweltsimulation.de/en/products/climate-c.html
Linear unit	*LES4 / IT116* (isel Germany AG, Eichenzell, Germany)	1	€ 1835.10	€ 1835.10	https://www.isel.com/de/produkte/mechanik/lineareinheiten/lineareinheit-les4.html
Balance	AR 0.6 kg / LCV-U5 (Lorenz Messtechnik GmbH, Alfdorf, Germany)	1	€ 306.78	€ 306.78	http://www.lorenz-messtechnik.de/deutsch/produkte/plattform_w/w-ar.php
AD-Unit – Balance and velocity control	USB-6002 (National Instruments Germany GmbH, München, Germany)	1	€ 349.15	€ 349.15	http://www.ni.com/de-de/support/model.usb-6002.html
AD-Unit – balance tempering	USB-6000 (National Instruments Germany GmbH, München, Germany)	1	€ 113.05	€ 113.05	http://www.ni.com/de-de/support/model.usb-6000.html
Vane anemometer	1468 (LAMBRECHT meteo GmbH, Göttingen, Germany)	1	€ 968.45	€ 968.45	http://www.lambrecht.net/datasheets/stroemung/1468_prospekt_de.pdf
Fan	8212J/2H4P (ebm-papst Mulfingen GmbH & Co. KG, Mulfingen, Germany)	1	€ 63.07	€ 63.07	http://www.ebmpapst.com/de/products/compact-fans/axial-compact-fans/axial_compact_fans_detail.php?pID=111780
USB <> RS232 Interface	“USB <> RS232 Interface Cable 2” (Wiesemann & Theis GmbH, Wuppertal, Germany)	2	€ 55.40	€ 110.80	https://www.wut.de/e-38011-ww-daus-000.php
Load resistor	RH-5–1%-15R (Vishay Electronic GmbH, Selb, Germany)	3	€ 2.76	€ 8.28	https://www.buerklin.com/de/praezisions-hochlast-drahtwiderstand/p/63e362
NTC	TS-NTC-103 (B + B Thermo-Technik GmbH, Donaueschingen, Germany)	4	€ 3.93	€ 15.72	https://shop.bb-sensors.com/Messtechnik-je-Branche/Maschinenbau/Praezisions-Temperatursensor-TS-NTC-103–10-k.html?listtype=search&searchparam=temperatursensoren
Mosfet	IRLZ24NPBF (Infineon Technologies, Neubiberg, Deutschland)	2	€ 0.39	€ 0.78	https://www.conrad.de/de/p/infineon-technologies-irlz24npbf-mosfet-1-hexfet-45-w-to-220–162869.html
thermal switch	TK24-T01-MG01-Ö75-S65 (Thermorex, Paris, France).	1	€ 2.69	€ 2.96	https://www.conrad.de/de/p/thermorex-tk24-t01-mg01-oe75-s65-bimetallschalter-250-v-16-a-oeffnungstemperatur-5-c-75-c-schliess-temperatur-65-c-1–1678259.html
PWM Controller	TL494 CN Pulse-Width-Modulation Control Circuits	1	€ 0.82	€ 0.82	http://www.ti.com/lit/ds/symlink/tl494.pdf
Control Unit	Personal Computer, Microsoft Windows 7	1	€ ~500	€ 500	
Total				€ ~40,000	

### 5 Build introductions

#### 5.1 Drying unit (II)

For assembling the drying unit, you have to build all parts described in the related design files. The plastic parts can either be lathed or produced in a 3D printer.

First, weld parts (7), (8) and (10) together. It is necessary to work precisely to obtain a concentric alignment. To avoid a distortion of the material, it is advisable to weld the parts only with spots and to close the inner edges afterwards with silicone.

The same procedure follows for parts (6), (7), (12) and also for (9) with two times (11).

Screw the lower part of (6) together with the cylindrical part of part (9) using screws (27) and hex nuts (1). All screws should be tighten hand-tight.

In the next step, assemble the outflow parts (14) and (17) by using screws (28) and distances (15).

To obtain the possibility of leveling the drying unit, screws (16) are mounted and countered with the hex nuts (1).

Assemble now the lower part of the measurement unit (around part 6) through the fan (5) on the top of the outflow unit (17) and use the screws (28).

Press part (19) on top of part (8). If the fit is not tight, silicone can be used to obtain the tight fitting of the components.

Assemble the sample holder with the parts (20), (21) and (25).

Glue the sieve (22) into the plastic cylinder (18).

After drilling the hole for the nylon strings in (4), install the screws (4) into the truss (23) up to half of the thread length.

The nylon strings (3) need to be cut to the necessary length. Either fix the nylon string at the upper end of the screw (4) with knots or glue it. Guide the string through the screws and fix the lower end of the nylon string at part (21) in the same way as the upper end. Repeat the procedure for all three strings.

Insert the vane anemometer (13) into the outflow unit, consider the right alignment while mounting. Afterwards, fit the upper part of the measurement unit on the vane anemometer and further install the sample holder on part (20).

In case of constructional leakages, silicone can be used for sealing the system.

After finishing the construction, tighten all screws carefully to avoid damages to the threads.

Place the drying unit in the air conditioning unit and prepare part (24) in the truss for later assembling.

#### 5.2 Balance unit (III)

Assemble the linear spindle drive consisting of parts (1), (2), (3) and (4).

Mount the strut to the linear spindle drive.

Mount the load resistors on the cooling unit and connect them according to the wiring diagram. Prepare the connection cords for connecting the electronic parts to the control unit. Afterwards, place the NTC and clue them to the certain positions. Fix the cooling unit (12) with screws and hex nuts (15 and 16) at the bottom of the top plate (11).

Assemble the lower part of the balance unit, parts (8), (9), (10) and (13) with screws (14).

Mount the balance (7) on top of part (9) with the screws (17). If necessary, place washers between balance and bottom plate. Drill a hole concentrically through the screw for preparing (6). Screw (6) halfway into the load cell.

Mount the balance unit on top of the strut and tighten the screws (18).

Lead all cables through the holes in the back plate (13) and close the unit with part (11) and the appropriate screws.

Fix the complete unit on top of the air conditioning unit, and align both of them in a way that the load cell is precisely above the opening of the climate chamber.

Open the top and connect the load cell through the bore in the climate chamber with the truss of the drying unit. Furthermore, fix the nylon string either with knots or glue. Close the balance unit again.

Carefully seal the opening of the climate chamber with a heat insulating material and make sure that the string does not touch the wall or the heat insulating material.

Make sure that the string is long enough, that the spindle drive can reach the upper position without tearing the string. At each start-up, the spindle drive has to reach the reference points for initialization.

#### 5.3 Commissioning of the system

Assemble electrical parts as described in the electrical circuits and connect them to the PC or the control cards.

Check the communication of all systems with the controller.

When all systems are responding, drive up and down the spindle unit and check the sample holder. Level it with the screws.

For providing reliable results, calibrate the full system. For calibration follow the description provided in the following chapter. If results of the calibration procedure are stable, the system is ready for use.

### 6 Operation instructions

#### 6.1 Operating procedure

**Fig. 5:**
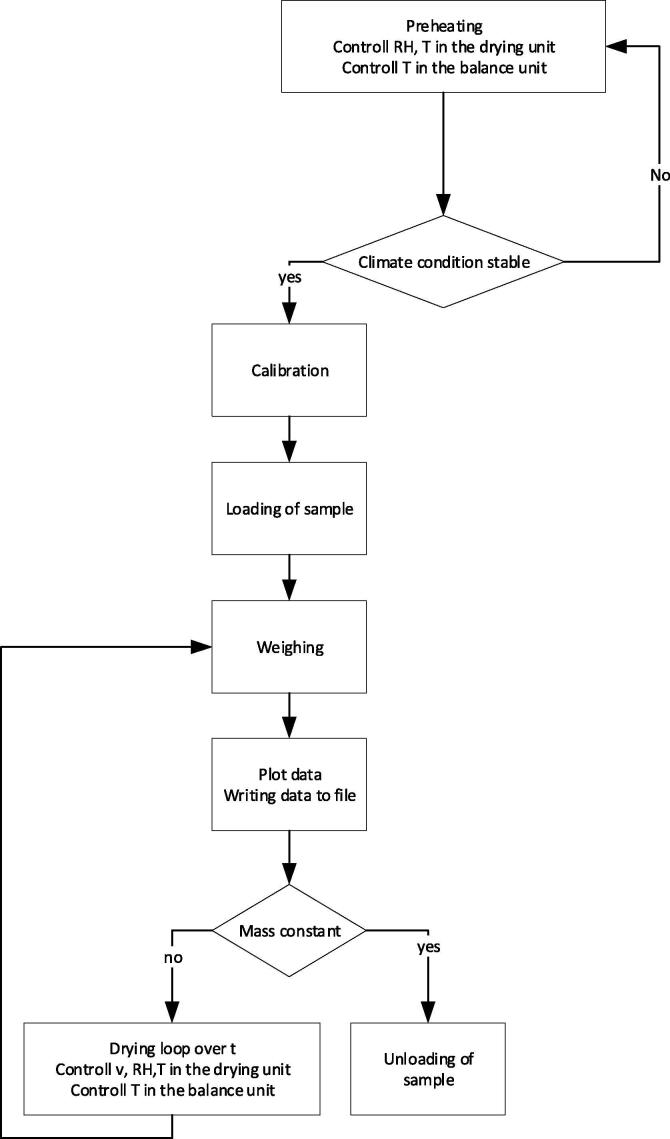
Operating procedure of the high precision laboratory dryer HPD TF1.

The high precision laboratory dryer HPD TF1 is operated by a procedure supported by a specially developed LabVIEW program. The process sequence is shown in Fig. 5.

A standby loop to preheat the drying unit and the balance unit is started when the HPD TF1 is activated. The condition of the air in terms of T and RH in the drying unit is displayed in a diagram on a control monitor, where T in the balance unit (III) and the course of measured mass is also plotted. As soon the drying air condition has stabilized at the set values, the mass calibration of the empty sample holder begins. After calibration and loading the probe, the main drying loop is started with an initial weighing of the probe. After lowering the sample holder onto the drying column, the first drying loop starts.

**Fig. 6:**
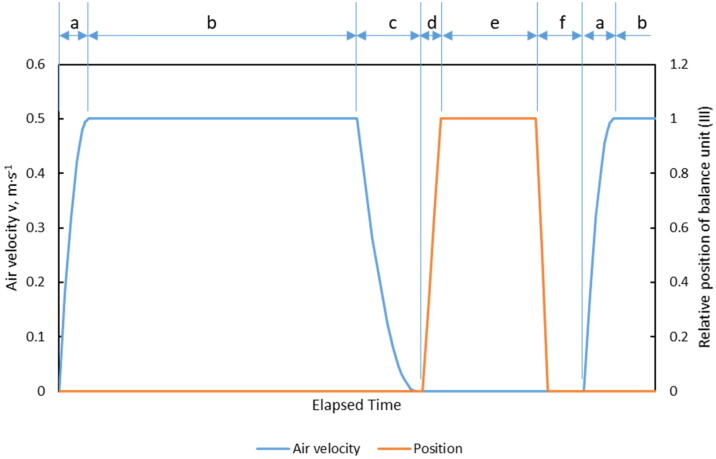
Coordinated operating mode of the fan and relative position of the balance unit over time during a drying loop of the HPD TF1; idealized curve.

The coordinated operating mode of the fan and balance unit during a drying loop is shown in Fig. 6.

In the initial phase (a), the fan starts and the rotation speed rises until the set air velocity is reached, which usually takes 5–10 s. Then the actual drying phase (b) starts, in which the product is aerated in a uniform through-flow mode. Changes of flow resistance during drying are automatically compensated by controlling the speed of the fan. The condition of the air is continuously controlled by the air conditioning unit (I) during phases (a) and (b). When the set drying interval (typically between 5 and 30 min) is reached, the control system switches off the fan and the air conditioning unit during the inactive phase (c). The air velocity decreases to zero to prevent vibrations during subsequent lifting of the balance unit. The fan is totally stopped. The lifting phase (d) is started and the balance unit is raised until the sample holder lifts off the drying column and hangs freely. After reaching the upper end position and a short settling period, the mass of the sample is determined in the weighing phase (e). After weighing, the balance unit drops down in a lowering phase (f) to the lower end position and the control of the air velocity and the air conditioning is restarted. The overall time of reduced air velocity for the weighing procedure, i.e. phase (c) through phase (a) takes around 1 min. Air velocity during the drying phase (b) is averaged and recorded.

All measured parameters are recorded in a file for each drying loop and displayed on the graphical interface in order to monitor the drying process.

#### 6.2 Setting of the drying air condition

The air temperature range for the drying experiments could be set between 20 and 80 °C to protect electrical equipment from heat damage. The range of RH should set between 5 and 80 % and the range of the air velocity could be set between 0.3 and 2.0 m∙s^−1^.

To quantify the accuracy of the drying air condition control and the mass measurement, the mean absolute percentage error (MAPE) was calculated:

(1) $$\mathrm{MAPE}=\frac{100}{n}\sum_{t=0}^{n}\left|\frac{Y_{\mathrm{set}}\left(t\right)-Y_{\mathrm{meas}}\left(t\right)}{Y_{\mathrm{set}}\left(t\right)}\right|$$

where Y_set_(t) are the set or calibration values at time t and Y_meas_(t) are the measured values for m, T and RH at time t, respectively.

#### 6.3 Setting of the thermodynamic state of the drying air

To simulate drying air conditions of various climate zones or to dry by recirculating the drying air, where a different water content of the air occurs, respective set values RH_set_ were calculated as:

(2) $${\mathrm{RH}}_{\mathrm{set}}=\frac{P}{P_{w,sat}}∙\frac{X}{0.622+X}∙100$$

where X is the water content in kg water per kg of dry air (kg kg^−1^, i.e. de facto dimensionless), P is air pressure in kPa and P_w,sat_ is water vapor pressure at saturation in kPa, calculated according to Tetens [14]:

(3) $$P_{w,sat}={0.133∙10}^{\left(\frac{7.5∙T}{T+237.3}+0.66\right)}$$

The Tetens equation was used, as it represents very well the tabulated values in the relevant temperature range of 30 to 80 °C.

Air pressure is a function of the altitude of a location and can be calculated according to [15]:

(4) $$P=P_{0}∙e^{-\frac{z}{z_{P}}}$$

where P_0_ is the standard pressure at sea level with 101.325 kPa, z is the altitude in m and z_P_ is a constant with 7290 m.

**Fig. 7:**
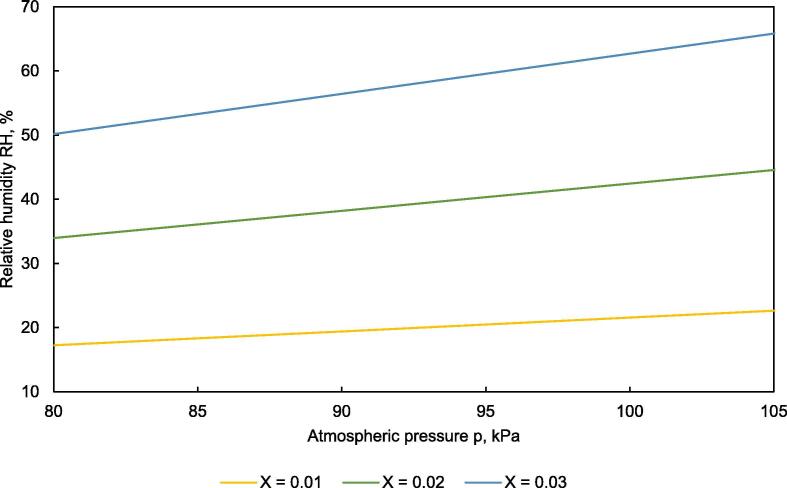
Relative humidity vs. atmospheric pressure for different water contents X of air at a set temperature T_set_ = 40 °C.

Fig. 7 shows RH versus air pressure for different water contents of the air at 40 °C. The gradient for RH increases with increasing water content and is 0.63 % (RH) per kPa for X = 0.03. At an atmospheric pressure of 80.0 kPa, representing an altitude of 1700 m, RH_set_ would be 50 % compared to 63 % for 101.3 kPa at sea level. Therefore, the altitude should be considered in the calculation of RH_set_. In contrast, pressure fluctuations due to weather conditions typically show an amplitude smaller than 0.5 kPa, where the effect on RH is negligible.

#### 6.4 Calibration of the vane anemometer

The vane anemometer of the dryer column is designed for measurements in free airflow. Therefore, a calibration is needed to adjust the anemometer to the forced airflow given by the experimental setup. To ensure a uniform airflow in the measurement section, the inflow section of the dryer column was enlarged to a length of 1100 mm, which is equal to 15 times of its diameter. This is within the recommended range for inflow of 10–20 diameter equivalents [16]. The range of the air velocity for calibration was set at 0.8–8 m·s^−1^, related to the free cross section of the sample holder of the drying column. An air density of 1.2 kg·m^−3^ and a dynamic viscosity of 15·10^−6^ m^2^·s^−1^ were taken as standard condition [16]. The hydraulic diameter of 72 mm is equal to the inner diameter of the measurement tube. Reynolds number was in a range of 9600–48,000 for a velocity range of 2–8 m·s^−1^, which means that conditions for a turbulent airflow are given [16].

For calibration, air velocity was measured at different rotational speeds of the fan by inserting a hot wire anemometer (Nr. 0635 1025, Testo AG, Lenzkirch, Germany) through a bore in the tube extension in an orthogonal direction to the flow. Measured values were logged with a multifunction meter (Type 435-4, Testo AG, Lenzkirch, Germany) once per second. Average air velocity over the cross section was calculated as 0.807· v_max_ [16], based on the maximum velocity, measured in the axis of the measurement tube.

**Fig. 8:**
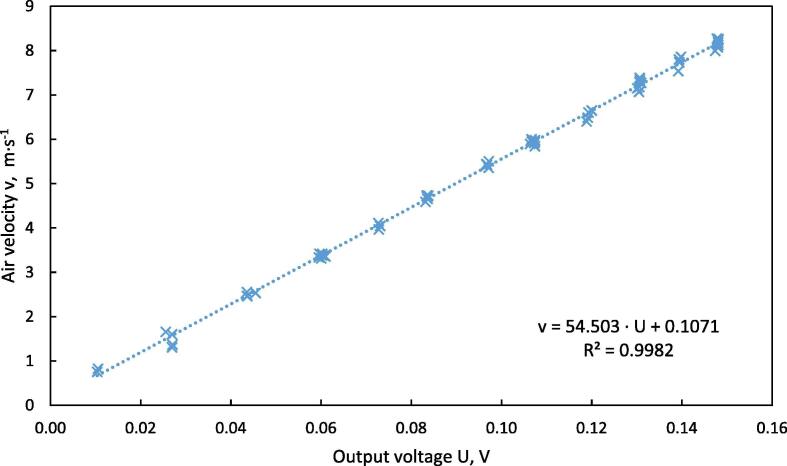
Calibration curve of the vane anemometer, air velocity vs. output voltage.

Simultaneously, the voltage output of the vane anemometer was measured via a shunt resistant using the I/O device USB-6002. The PWM-Signal was controlled by a LabVIEW routine in 10 % intervals. Once the duty cycle was set to a specific value the software waits for a stable 5 s period of the vane anemometer signal. The average voltage of the stable period was recorded and compared to the measurements of the heat wire anemometer. The scatter plot is shown in Fig. 8, the values were fitted at R^2^ = 0.9982 by a linear function shown in Eq. (5):

(5) v=54.503∙U+0.1071

### 7 Validation and characterization

#### 7.1 Signal stability and mass drift compensation of the balance

**Fig. 9:**
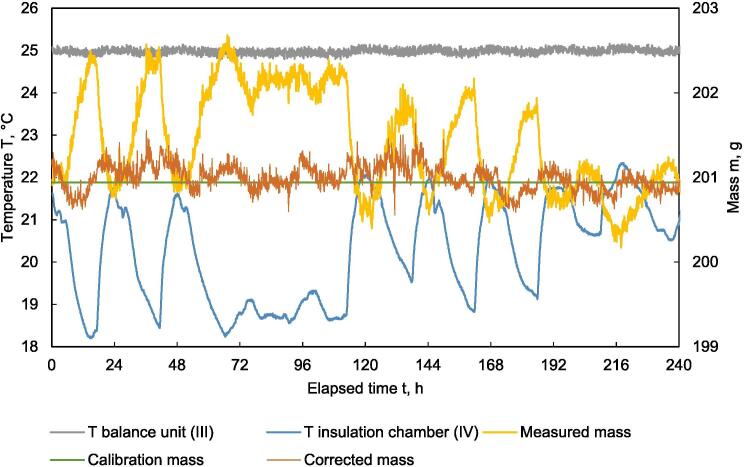
Temperature and mass vs. time; temperature in thermal insulation chamber (IV), temperature in balance unit (III), measured mass m_meas_, calibration weigth m_cal_ and corrected mass m_corr_ in the HPD TF1 during calibration.

Electronic balances based on load cells are known for their sensitivity to temperature influences, therefore the zero drift must be corrected accordingly. To measure the mass drift, a weight with a known mass of 200.94 g was placed in the sample holder serving as dead load. Fig. 9 exemplarily shows the course of the different temperatures during ten days. The temperature in the thermal insulation chamber (IV) showed diurnal fluctuations with a range of 18.2 to 22.3 °C. The two lower temperature peaks show weekend days where the building was not heated. The temperature inside the balance unit (III) showed fluctuations in a small range of 24.8 to 25.2 °C, which indicates that the thermostatic control was working well, which is also affirmed by a MAPE of 0.24 %.

**Fig. 10:**
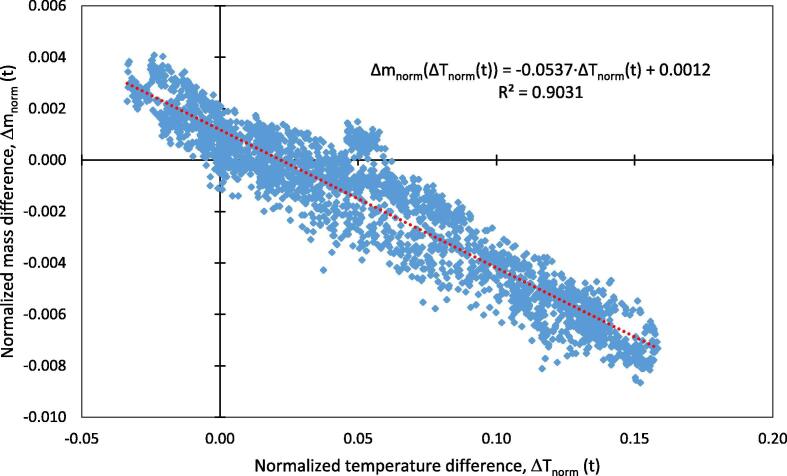
Normalized mass difference vs. normalized temperature difference in the HPD TF1 during calibration.

Furthermore, Fig. 9 shows the course of mass with the dead load of 200.94 g. The range of mass fluctuation was 2.56 g. A MAPE of 0.28 %, calculated for a period of 240 h, shows a good signal stability of the balance. The daily fluctuation of the temperature is reflected in the mass value. The normalized deviation of the mass difference versus the temperature difference in the HPD TF1 as calculated according Eqs. (6), (7), is shown in Fig. 10. The lifting of the balance inevitably causes vibrations, which are reflected in the signal. The scatter plot for normalized deviation of mass versus temperature shows a linear trend with R^2^ = 0.9031 (Fig. 10). Eq. (8) of the respective regression line is used for mass drift compensation using Eq. (9). Corrected mass (m_corr_) after compensation is also shown in Fig. 9. Minimum and maximum mass values and MAPE are presented in Table 1. The corrected signal shows a better result than the raw signal. Comparing MAPE from the raw signal m_meas_ with the corrected signal m_corr_, the MAPE is reduced from 0.28 % to 0.07 %. This shows that the weighing can be improved by temperature compensation. However, the mass drift is very low even without compensation and high precision measurements can be performed without mathematical correction.

**Table 1:** Measured and corrected values m_meas_ and m_corr_ for dead load m_cal_ together with MAPE during calibration of the HPD TF1.

m_cal_ g	m_meas_ min g	m_meas_ max g	m_corr_ min g	m_corr_ max g	MAPE (m_meas_) %	MAPE (m_corr_) %
200.94	200.12	202.68	200.34	201.64	0.28	0.07

**Fig. 11:**
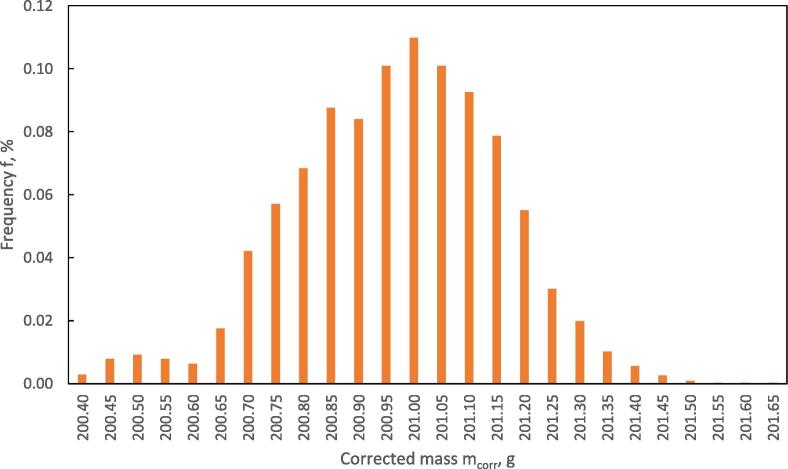
Frequency of corrected mass vs. corrected mass in the HPD TF1 during calibration with a time interval Δt = 300 s between the measurements, n = 3013.

The statistical evaluation of the mass calibration shows a very good distribution of the measuring points after the calculation of the corrected values m_corr_. The distribution is shown in Fig. 11. The standard deviation and standard error over the measurement period with 3013 measurements shows very low error values, shown in Table 2.

(6) $${\Delta m}_{norm}\left(t\right)=\frac{m_{meas}\left(0\right)-m_{meas}\left(t\right)}{m_{meas}\left(0\right)}$$

(7) $${\Delta T}_{norm}\left(t\right)=\frac{T_{meas}\left(0\right)-T_{meas}\left(t\right)}{T_{meas}\left(0\right)}$$

(8) $$\Delta m_{norm}\left(t\right)=-0.0537∙{\Delta T}_{norm}\left(t\right)+0.0012$$

(9) $$m_{corr}\left(t\right)=m_{meas}\left(t\right)+\Delta m_{norm}\left(t\right)∙m_{meas}\left(0\right)$$

(10) $$u=\sqrt{\sum_{i=1}^{n}u_{i}^{2}}$$

**Table 2:** Statistical evaluation of the mass calibration.

	n	m_average_	m_cal_	Standard deviation	Standard error of the mean value
m_corr_	3013	200.94	200.94	0.18630	0.00339

Reproduction law for measurement uncertainties according to Gauss is calculated with Eq. (10) [17]. For the mass balance a measurement uncertainty is calculated of ±0.12 %. The Calibration shows, that the measured values are in a trustable range.

#### 7.2 Velocity of drying air over time

In order to validate the stability of the air velocity over time, test runs were performed with different set values for air velocity v_set_, which were 0.3, 0.5 and 0.7 m·s^−1^. To include the effect of changing the airflow resistance of the material bulk during drying, separated solids biogas digestate was filled 80 mm high into the sample holder. Drying temperature was set to T_set_ = 60 °C and relative humidity to RH_set_ = 15 %.

**Fig. 12:**
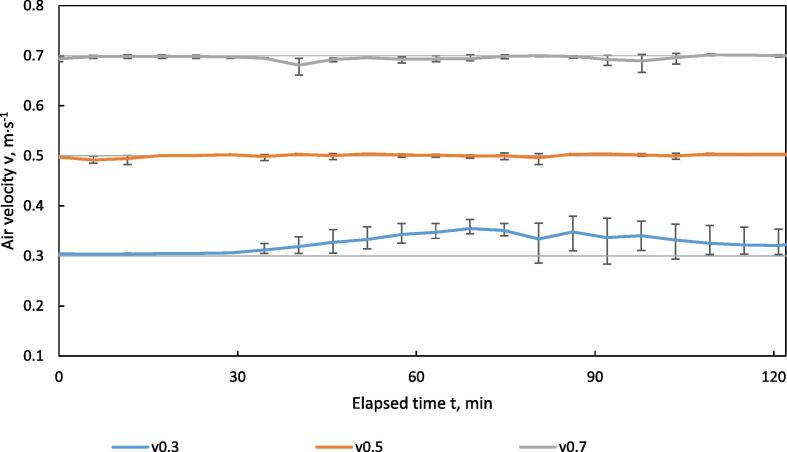
Air velocity of drying air vs. time in the HPD TF1 during drying of separated digestate at different levels of set air velocity v_set_ = 0.3_,_ 0.5, 0.7 m·s^−1^; T = 60 °C, RH = 15 %, n = 3; error bars indicate min/max-range.

Fig. 12 shows the course of the measured air velocity v_meas_ over time. For the higher set values v_set_ of 0.7 and 0.5 m·s^−1^, the air velocity could be kept stable by the control system during the entire drying process. However, at the lowest value set at 0.3 m·s^−1^, the air velocity could not be reduced sufficiently to react to the decreasing airflow resistance of the bulk material during drying, as the minimum speed of the fan was reached. The disturbed control process also caused higher fluctuations and a higher MAPE of 8.91 % compared to 0.63 % and 0.55 % for 0.5 and 0.7 m·s^−1^, respectively (Table 3).

**Table 3:** Min/max value and MAPE for air velocity of the drying air in the HPD TF1 during drying of separated digestate at different set values.

v_set_ m·s^−1^	v_meas_ min m·s^−1^	v_meas_ max m·s^−1^	MAPE (v) %
0.3	0.28	0.38	8.91
0.5	0.48	0.51	0.63
0.7	0.66	0.70	0.55

#### 7.3 Temperature of drying air over time

To validate the temperature stability over time, test runs with different set values of 40, 60 and 80 °C for the drying air temperature T_set_, were conducted. Water content of the drying air X was set to 0.02 resulting in a relative humidity of the drying air RH_set_ of 42.7, 15.8 and 6.7 %. Air velocity was set at 0.5 m·s^−1^.

**Fig. 13:**
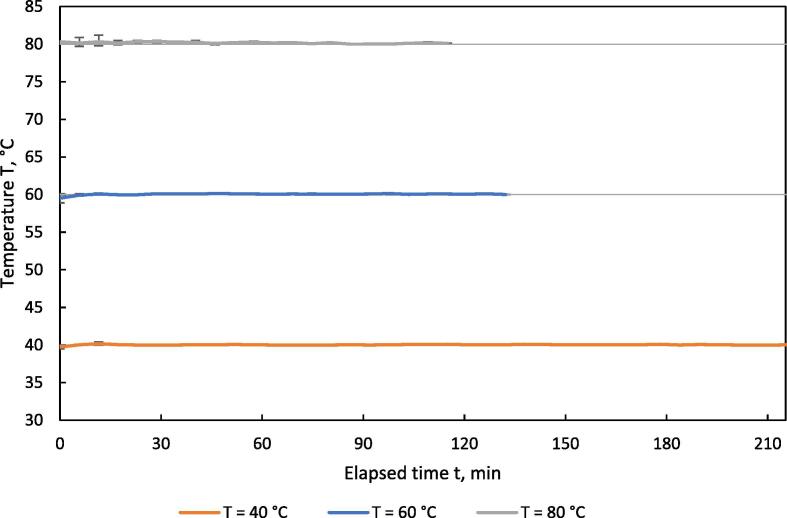
Temperature of drying air vs. time in the HPD TF1 during drying of separated digestate at different levels of temperatures; n = 3, v_set_ = 0.5 m·s^−1^, X_set_ = 0.02; error bars show min/max-range.

Fig. 13 shows the course of the measured drying air temperature T_meas_ over time. The temperature was only slightly below the set values at the beginning of the test runs when the door was closed. Hardly any fluctuations occurred in the further course, which is also documented by the low MAPE below 0.21 %, see Table 4.

**Table 4:** Min/max value and MAPE for the temperature of the drying air in the HPD TF1 during drying of separated digestate at different set values.

T_set_ °C	T_meas_ min °C	T_meas_ max °C	MAPE (T) %
40	39.5	40.1	0.11
60	58.9	60.2	0.14
80	79.7	81.2	0.21

#### 7.4 Relative humidity of the drying air over time

To validate the stability of the relative humidity of the drying air over time, test runs were performed with different set values for the water content X_set_, which were 0.01, 0.02 and 0.03. Temperature was set to T_set_ = 40 °C resulting in a relative humidity of the drying air, RH_set_ = 20.5, 40.5 and 59.8 % based on a pressure of p_w,sat_ = 95.6 kPa for the location Stuttgart-Hohenheim (420 m a.s.l.). Air velocity was set to v_set_ = 0.5 m·s^−1^. To include the effect of evaporating water from the material bulk during drying, separated solid biogas digestate was filled 80 mm high into the sample holder.

**Fig. 14:**
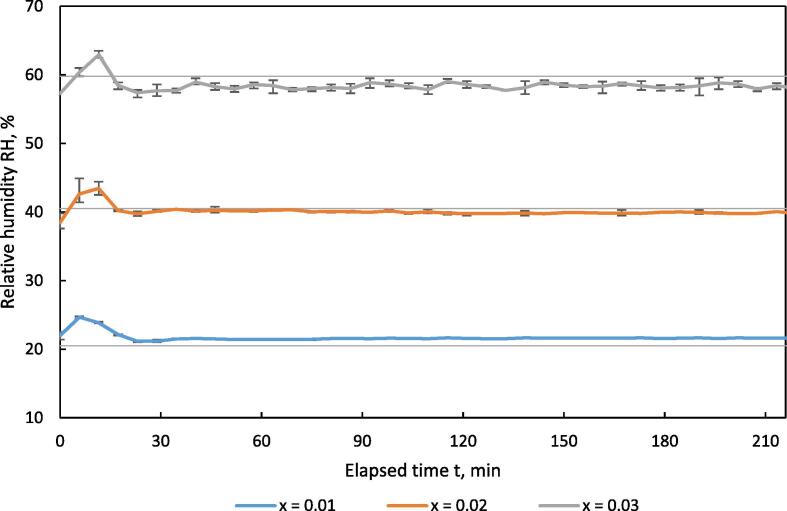
Relative humidity RH of the drying air vs. time t in the HPD TF1 during drying of separated digestate at different levels of water content X of the drying air; n = 3, v = 0.5 m·s^−1^, T = 40 °C; error bars show min/max-range.

Fig. 14 shows the course of the measured relative humidity RH_meas_ of the drying air over time. Fluctuations occur in the first minutes after starting a test run. First, RH_meas_ falls below the set value because the door of the drying chamber was opened to load the sample holder. The control program of the climate chamber detects a negative difference in the RH and starts to humidify the chamber. At the same time, the drying of the product starts with an evaporation of around 2 g of water per 5 min. The positive difference in the RH initiates the dehumidifying process and a slight overshooting can be detected. After 20 min, the climate condition was established according to the set values and the course of the RH remained constant over the remaining drying cycle.

For calculating the actual water content X_calc_ from measured data, Eq. (11) was converted to:

(11) $$X_{\mathrm{calc}}=0.622∙\frac{\frac{{\mathrm{RH}}_{\mathrm{meas}}}{100}∙P_{w,sat}}{P-\frac{{\mathrm{RH}}_{\mathrm{meas}}}{100}∙P_{w,sat}}$$

where RH_meas_ is the measured relative humidity and P_w,sat_ is calculated for the measured temperature T_meas_.

**Fig. 15:**
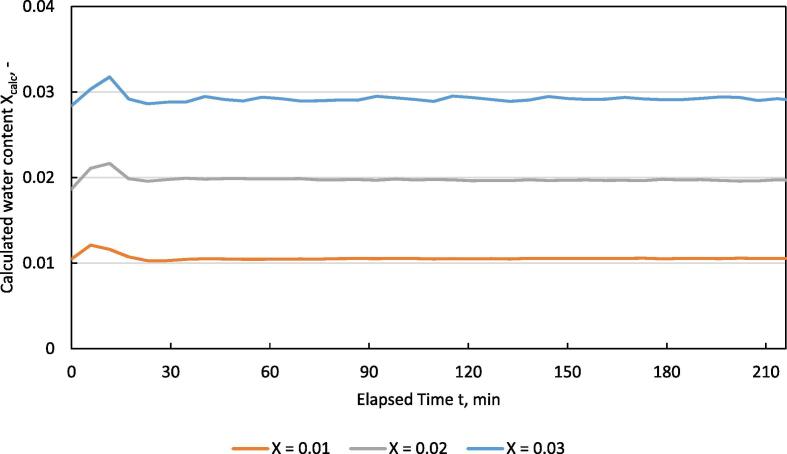
calculated water content of drying air vs. time in the HPD TF1 during drying of separated digestate at different water content levels X of the drying air; n = 3, X_calc_ calculated out of T_average_ and RH_average._

Fig. 15 shows the course of the water content of the drying air X_calc_ over time.

Results for MAPE are shown in Table 5. MAPE for the RH indicates larger differences between the single trials. The absolute error fluctuates by 5 %, which has a more pronounced effect on the MAPE at lower set values for RH. The calculated MAPE for water content of the drying air X_calc_ indicates a high level of stability. In addition, the absolute deviation in the water content X between the minimum and maximum values shows only a small difference. The stability of the system is well suited for drying experiments.

**Table 5:** MAPE for temperature, relative humidity and calculated water content of the drying air in the HPD TF1 during drying of separated digestate; T_set_ = 40 °C.

X_set_	RH_set_ %	RH_meas_ min %	RH_meas_ max %	X_calc_min	X_calc_max	MAPE (RH) %	MAPE (X) %
0.01	20.5	21.0	24.8	0.0103	0.0121	5.47	3.69
0.02	40.5	37.6	44.9	0.0187	0.0217	1.66	3.43
0.03	59.8	55.1	63.5	0.0284	0.0318	2.06	4.40

#### 7.5 Mass loss of the drying material over time

To validate the measurement of mass loss of the drying material over time, test runs were performed at v_set_ = 0.5 m·s^−1^, T_set_ = 40 °C and RH_set_ = 20.5, 40.5 and 59.8 %. Separated solid biogas digestate was filled 80 mm high into the sample holder. Three replicates were performed per RH setting.

**Fig. 16:**
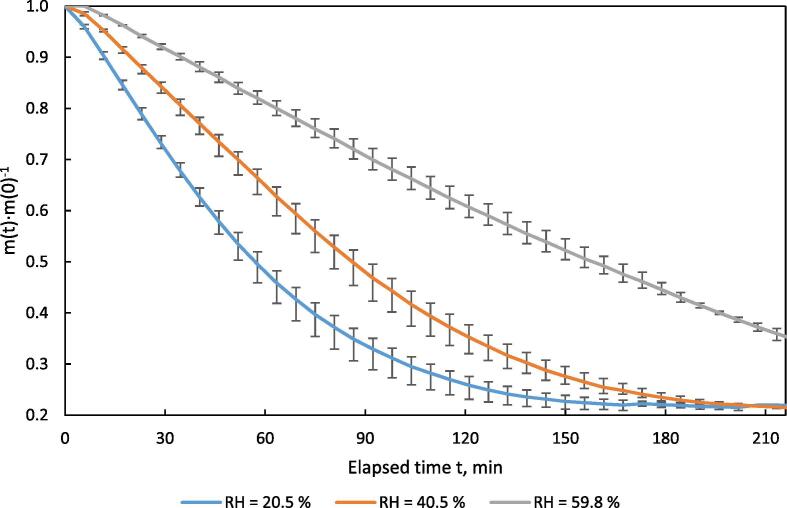
Mass vs. time in the HPD TF1 during drying of separated digestate at different water contents of the drying air; n = 3, v = 0.5 m·s^−1^, T = 40 °C; error bars show min/max-range.

The course of the relative sample mass for different levels of relative humidity is displayed in Fig. 16. As expected, the mass loss rate was lower at higher levels of humidity. Although the different water contents of the drying air have an influence on the drying time, this has no negative effect on the uniformity of the drying process itself. Especially at the beginning and at the end the error bars are small, which indicates a high accuracy of the dryer and shows that the results of the drying tests are reproducible. Larger error bars in the middle phase are related to the inhomogeneity of the drying rate, which was also found in other studies [11].

### Funding

The study was performed within the project GOBi funded by the Federal Ministry of Education and Research (BMBF), represented by the Project Management Jülich, under grant number 03EK3525A.

## Declaration of Competing Interest

The authors declare that they have no known competing financial interests or personal relationships that could have appeared to influence the work reported in this paper.
